# Women attending the sexual assault treatment unit services in the Republic of Ireland: A 7‐year review

**DOI:** 10.1002/ijgo.15947

**Published:** 2024-10-09

**Authors:** D. Kane, J. Walshe, N. Maher, C. Pucillo, D. Richardson, A. Holmes, K. Flood, M. Eogan

**Affiliations:** ^1^ Department of Obstetrics & Gynaecology Royal College of Surgeons in Ireland Dublin Ireland; ^2^ Sexual Assault Treatment Unit Rotunda Hospital Dublin Ireland; ^3^ Sexual Assault Treatment Unit Galway Ireland

**Keywords:** rape, sexual assault, sexual health, violence, women's health

## Abstract

**Objective:**

Sexual assault is pervasive in today's society, with the numbers of those reporting it increasing. In Ireland, 50% of women will experience some form of sexual violence in their lifetime. We sought to describe the incident details of females presenting to the Sexual Assault Treatment Unit (SATU) network in the Republic of Ireland and to determine associations between incident characteristics and: (1) victim age, (2) presence of injury, (3) victim‐perpetrator relationship, and (4) number of assailants.

**Methods:**

This was a retrospective cross‐sectional study of all females who attended between 2017 and 2023. Descriptive bivariate analysis was performed.

**Results:**

There were 5942 female attendances, with an average age of 26 years. The largest age group was women between 18 and 25 years (38.1%, *n* = 2263). Forensic examinations were performed in 76.6% (*n* = 4549). Assailants were male in 92% (*n* = 5469) of incidents, with multiple assailants disclosed in 7.3% (*n* = 435). Strangers or recent acquaintances were the assailant in 38.5% (*n* = 2290) of incidents, and close associates in 22.9% (*n* = 1359). Incidents occurred at the survivor's home in 22% (*n* = 1306) of incidents, and in the assailant's home in 22.6% (*n* = 1342). Drug use within 24 h was reported in 15.1% (*n* = 897) of cases, and alcohol use in 72% (*n* = 4276). Drug‐facilitated assault was suspected by 16.1% (*n* = 955). Injuries (genital and extra‐genital) occurred in 30.3% (*n* = 1800) of attendances and were more likely to be seen in those who disclosed ingesting alcohol (Relative risk [RR] 1.325, *P* < 0.001) or drugs (RR 1.111, *P* = 0.04) in the 24 h preceding the incident, in those who presented within 24 h (RR 1.646, *P* < 0.001), those aged 18 years or older (RR 1.07, *P* = 0.003), and those where the incident occurred outdoors (RR 1.24, *P* < 0.001).

**Conclusion:**

This study, one of the largest on female attendances to a national SATU network, offers detailed insights into demographics, incident details, and circumstances. Most were young women, mainly 18 years and older, many of whom were full‐time students. Forensic examinations were the primary reason for attendance, underscoring the network's key role in evidence collection. The study also identified factors linked to a higher risk of injury detection, such as immediate post‐incident attendance, being over 18, outdoor incidents, perpetration by a stranger, and prior alcohol or drug use.

## Synopsis

This study analyzed 5942 female attendances at Sexual Assault Treatment Units in Ireland (2017–2023), identifying patterns and associations with age, injury, victim–perpetrator relationship, and number of assailants.

## 1 INTRODUCTION

Sexual assault is prevalent in today's society, with 50% of women in Ireland disclosing a history of sexual violence in their lifetime.^1^ Sexual violence is considered any unwanted sexual activity, ranging from non‐contact experiences to non‐consensual sexual intercourse. It is a pervasive issue affecting individuals worldwide, with potentially profound physical, psychological, and social consequences for survivors.^2^, ^3^ Recognizing the multifaceted needs of people who experience sexual assault in the Republic of Ireland, Sexual Assault Treatment Units (SATUs) were established as specialized facilities dedicated to providing streamlined comprehensive care and support.^4^ SATUs provide medical, forensic and psychological support to victims of sexual assault.

The development of SATUs marked a significant advancement in addressing the immediate and long‐term needs of sexual assault victims. The national SATU network in Ireland provides care through six units, spread geographically throughout the country with standardized national guidelines, database, training, and robust interagency links.

Having comprehensive national data on sexual assault unit attendances is invaluable for several reasons. Such data allow for a richer understanding of the scope and scale of the issue. This macro‐level perspective is crucial for policymakers and healthcare providers to allocate resources effectively, ensuring that support services are sustainable, adequately funded, and accessible to all regions. The data are also valuable for individual SATUs and allied agencies, enabling each service to compare themselves against national metrics, and advocate for appropriate resources to meet service need. The findings may also be relevant for similar services internationally, while also being useful for education and discussion. National statistics also help to identify trends and patterns that might not be apparent at a local level, providing insights into regional similarities and variations and the potential underlying causes of these differences.^5^, ^6^ Therefore, the SATU service was motivated to collate, analyze, and report these metrics from the national SATU network in the Republic of Ireland.

The main objectives of this study are to provide detailed descriptive data on females who disclose sexual assault, analyze the circumstances surrounding these assaults, and identify risk factors that can guide the development of preventive measures. Specifically, the study aims to explore the relationships between the person's age, their relationship with the perpetrator, and the circumstances of the assault. Additionally, it seeks to assess the presence of injuries and compare incidents involving multiple assailants with those involving a single assailant. The study's findings aim to identify patterns which might inform mitigation strategies, such as whether younger women are at increased risk in specific environments or if assaults are more prevalent when the perpetrator is known to the victim. These insights are intended to inform the development of public health strategies, educational campaigns, service enhancements, and support tailored responses from An Garda Síochána (police) and other support services.

## 2 METHODS

### 2.1 Study design

This was a retrospective cross‐sectional study analyzing the attendances of all female patients at the national SATU network between 2017 and 2023. The inclusion criteria for this study were all female attendances to the SATUs during the study period. Attendances were defined as the first visit to the SATU after a sexual assault. This was either for a sexual health screen or for a forensic examination. Exclusion criteria were anyone who did not identify as female, those who did not disclose a sexual assault, or did not disclose that they were concerned a sexual assault might have happened.

### 2.2 Ethical approval

Ethical approval for this study was sought and granted from the Research Advisory and Guideline Group, Rotunda Hospital, Dublin 1, Ireland (RAG‐2022‐005).

As the data analyzed were irrevocably anonymized, individual patient consent was not required. Of note, each patient attending a SATU is asked to sign a consent form at the end of their visit, to allow their data to be used for research purposes. Participation is voluntary and patients may choose to decline.

### 2.3 Study setting and population

In the Republic of Ireland, the SATU network comprises six units providing 24/7 care for individuals of all genders aged 14 years and over who disclose sexual assault. Occasionally, SATUs treat those under 14 when pediatric services are unavailable. Care is standardized and guided by national evidence‐based guidelines, regularly updated with international best practices, and evaluated through audits, peer reviews, and performance monitoring. SATUs address medical, psychological, and emotional needs while also providing forensic examination to aid criminal investigation. Forensic exams are conducted by specially trained doctors or nurses, with patients choosing between police involvement, health‐check only (sexually transmitted infection screening), or forensic examination without immediate police involvement (evidence storage). For those who choose a forensic examination without immediate police involvement, the forensic samples are securely stored in the SATU for a period of 1 year. If a person chooses to report the crime to the police, the samples can be released with the patient's consent. Otherwise, the samples are destroyed. Forensic examinations are guided by the survivors' disclosure and preference, and include comprehensive physical and ano‐genital examinations, using sterile speculums and proctoscopes as needed. Forensic samples (DNA and toxicology samples) are collected based on the type of assault and time elapsed. Colposcopy and genital photo‐documentation are not currently used in the Irish SATU network. It is known that colposcopy increases the rate of detection of injury after both consensual and non‐consensual intercourse, particularly if it is carried out within 48 h of intercourse. There continues to be discussion around the evidential significance of ano‐genital findings at sexual assault forensic examination, and the increased identification of genital injury when colposcopy is used, as this does not precisely define the etiology of that injury or distinguish between consensual and non‐consensual contact.^7^, ^8^, ^9^

After each attendance, anonymized details are entered from the (paper) medical chart into a national database. The data that are recorded on this database include demographic details of the patient, incident (assailant–victim relationship, location, time of incident, etc.) and attendance details (type of attendance, day/time of attendance, time from incident to attendance, etc.). Injury data from the forensic examination (if applicable) are recorded as binary values (present/not present), with this metric including both genital and extra‐genital injury. This current data collection tool does not allow for disaggregation according to location of injury (i.e. genital or extra‐genital, or both).

### 2.4 Study protocol and data analysis

Irrecoverably anonymized patient demographic data as well as incident and attendance details were imported into Microsoft Excel from the national SATU database. For each of the variables, missing data were left blank. This occurred when data were not inputted into the database or when the forensic examiner did not ask or record the information. The quantum of missing data has been reported as a footnote of the tables accompanying this paper.

The data were then coded and imported into SPSS (version 26; SPSS, Chicago, IL, USA). Descriptive bivariate analysis was performed to study associations between various characteristics of the assault and age groups, as well as assailant–victim relationship. Associations as regards injury presence were also studied. We report numbers and percentages for descriptive statistics. The chi‐squared analysis was performed to compare relative frequencies. Odds ratios (ORs) and 95% confidence intervals (CIs) were also calculated; statistical significance was defined as *P*‐value <0.05, describing the probability that an association was due to chance.

## 3 RESULTS

### 3.1 Demographic details of attenders

**FIGURE 1:**
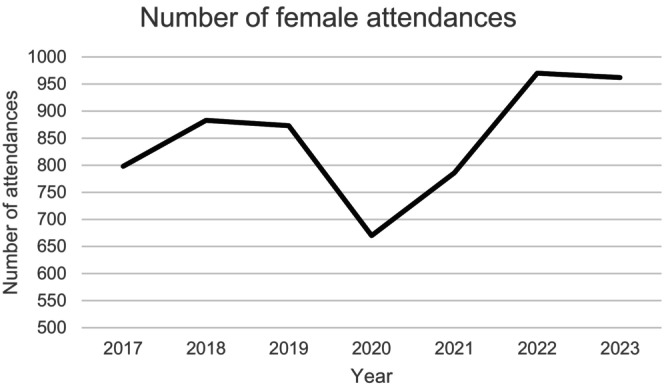
Female attendances at the Sexual Assault Treatment Unit (SATU) network by year.

During the study period, there were 5942 female attendances to the network, with the number of attendances per year shown in Figure 1. The mean age of attenders was 25.91 ± 11.46 years. Among them, individuals aged 18 and above constituted 4713 (79.3%) attendances, with those aged 18–25 years comprising 2263 (38.1%) of this group. Those in full‐time education represented 2266 (38.1%) of the attendees, while 1324 (22.3%) attendees were unemployed. Individuals with Irish nationality represented 4723 (79.5%) of female attendances, while those from other European nations constituted 506 (8.5%) attendances. Additionally, attendees included people from African (*n* = 182, 3.1%), South and Central American (*n* = 81, 1.4%,), and Middle Eastern (*n* = 28, 0.5%,) nations.

### 3.2 Attendance details

Forensic examinations were conducted in 4549 (76.6%) of attendances. Meanwhile, 1274 (21.4%) females attended for health checks, and 119 (2%) sought advice or other assistance. On‐site evidence storage was utilized in 701 (11.8%) attendances. The majority of attendances occurred within 7 days of the incident (*n* = 4540, 76.4%), with 2352 (39.6%) attendees presenting within the first 24 h. People were significantly more likely to attend within 24 h if the assailant was a stranger or someone known for less than 24 h (OR 1.74, 95% confidence interval [CI] 1.57–1.94, *P* < 0.001). As regards out‐of‐hours attendances (between 17:00 and 07:59), these accounted for 2607 (43.9%) attendances.

### 3.3 Incident details

In 5469 (92%) of cases, the assailant was male. Incidents involving strangers and recent acquaintances accounted for 2290 (38.5%) attendances, while those involving friends, intimate partners, or family members comprised 1359 (22.9%) attendances. Age‐specific characteristics of incident details are shown in Table 1. Incident characteristics stratified by victim–assailant relationship are shown in Table 2.

**TABLE 1:** Age‐specific characteristics of incident details.

	<14 y (*n* [%])	14–15 y (*n* [%])	16–17 y (*n* [%])	18–24 y (*n* [%])	25–34y (*n* [%])	35–44 y (*n* [%])	45–54 y (*n* [%])	55–70 y (*n* [%])	>70 y (*n* [%])
Total attendances by age group^a^	108 (1.8)	479 (8.1)	628 (10.6)	2263 (38.1)	1306 (22.0)	696 (11.7)	302 (5.1)	104 (1.8)	41 (0.7)
Location of incident^b^									
Home	31 (31.6)	50 (11.1)	59 (9.6)	390 (17.7)	348 (27.5)	238 (35.7)	128 (44.3)	46 (4.7)	12 (33.3)
Assailant's home	28 (28.6)	74 (16.5)	110 (18.0)	580 (26.3)	320 (25.3)	148 (22.2)	63 (21.8)	16 (16.5)	2 (5.6)
Other indoors	9 (9.2)	88 (19.6)	140 (22.9)	602 (27.3)	317 (25.0)	130 (19.5)	45 (15.6)	24 (24.7)	19 (52.8)
Field/park	8 (8.2)	94 (20.9)	88 (14.4)	79 (3.6)	37 (2.9)	18 (2.7)	10 (3.5)	2 (2.1)	0 (0.0)
Other outdoors	13 (13.3)	104 (23.2)	151 (24.7)	325 (14.7)	140 (11.0)	83 (12.5)	23 (8.0)	3 (3.1)	2 (5.6)
Taxi	0 (0)	1 (0.2)	1 (0.2)	32 (1.5)	21 (1.7)	7 (1.1)	2 (0.7)	0 (0.0)	0 (0)
Vehicle	6 (6.1)	28 (6.2)	45 (7.4)	93 (4.2)	46 (3.6)	18 (2.7)	9 (3.1)	4 (4.1)	0 (0)
Other	2 (2.0)	7 (1.6)	7 (1.1)	21 (1.0)	12 (0.9)	9 (1.4)	4 (1.4)	1 (1.9)	1 (2.8)
Unsure	1 (1.0)	3 (0.7)	11 (1.8)	83 (3.8)	26 (2.1)	15 (2.3)	5 (1.7)	1 (1.0)	0 (0)
Relationship of assailant to victim^c^, ^d^									
Acquaintance <24 h	5 (5.0)	59 (12.9)	93 (15.1)	428 (19.5)	201 (15.8)	82 (12.2)	29 (9.9)	9 (9.3)	1 (2.8)
Acquaintance >24 h	17 (16.8)	120 (26.3)	135 (22.0)	453 (20.7)	242 (19.0)	114 (17.0)	71 (24.1)	17 (17.5)	6 (16.7)
Family member	42 (41.6)	24 (5.3)	33 (5.4)	46 (2.1)	49 (3.9)	21 (3.1)	12 (4.1)	4 (4.1)	0 (0)
Intimate partner	2 (2.0)	17 (3.7)	28 (4.6)	72 (3.3)	94 (7.4)	76 (11.3)	35 (11.9)	16 (16.5)	1 (2.8)
Ex‐intimate partner	1 (1.0)	17 (3.7)	26 (4.2)	101 (4.6)	108 (8.5)	88 (13.1)	40 (13.6)	11 (11.3)	2 (5.6)
Friend	12 (11.9)	103 (22.6)	107 (17.4)	287 (13.1)	160 (12.6)	66 (9.8)	39 (13.3)	9 (9.3)	0 (0)
Stranger	11 (10.9)	81 (17.8)	152 (24.7)	605 (27.6)	306 (24.1)	159 (23.7)	45 (15.3)	12 (12.4)	9 (25.0)
Person in authority	4 (4.0)	4 (0.9)	7 (1.1)	27 (1.2)	21 (1.7)	16 (2.4)	5 (1.7)	4 (4.1)	7 (19.4)
Other	4 (4.0)	8 (1.8)	7 (1.1)	17 (0.8)	19 (1.5)	9 (1.3)	3 (1.0)	4 (4.1)	3 (8.3)
Unknown	3 (3.0)	23 (5.0)	27 (4.4)	156 (7.1)	71 (5.6)	41 (6.1)	15 (5.1)	11 (11.3)	7 (19.4)
Concern for a drug‐facilitated sexual assault^e^									
Yes	5 (6.5)	36 (8.5)	82 (14.0)	414 (20.0)	237 (19.9)	127 (20.5)	42 (15.7)	11 (12.5)	0 (0)
Unsure	7 (9.1)	49 (11.6)	90 (15.4)	364 (17.6)	197 (16.5)	102 (16.4)	44 (16.5)	12 (13.6)	4 (13.8)
No	65 (84.4)	337 (79.9)	412 (70.5)	1291 (62.4)	758 (63.6)	392 (63.1)	181 (67.8)	65 (73.9)	25 (86.2)
Drug use in the 24 h prior to incident^f^									
Yes	4 (4.9)	56 (12.7)	80 (13.3)	400 (18.6)	279 (22.6)	103 (15.7)	40 (14.2)	6 (6.4)	3 (10.0)
No	177 (95.1)	386 (87.1)	517 (86.2)	1741 (80.9)	950 (77.0)	545 (83.5)	239 (85.1)	86 (92.5)	27 (90.0)
Unsure	0 (0)	1 (0.2)	3 (0.5)	10 (0.5)	5 (0.4)	5 (0.8)	2 (0.7)	1 (1.1)	0 (0)
Alcohol use in the 24 h prior to incident^g^									
Nil	65 (78.3)	225 (50.0)	225 (37.3)	412 (19.3)	339 (27.7)	222 (34.2)	105 (37.6)	44 (47.3)	24 (85.7)
≤6 standard drinks	6 (7.2)	86 (19.1)	151 (25.0)	472 (22.1)	266 (21.7)	147 (22.6)	73 (26.2)	25 (26.9)	2 (7.1)
>6 standard drinks	11 (13.3)	131 (29.1)	224 (37.2)	1224 (57.2)	601 (49.0)	269 (41.4)	95 (34.1)	22 (23.7)	2 (7.1)
Unsure	1 (1.2)	8 (1.8)	3 (0.5)	32 (1.6)	20 (1.6)	12 (1.8)	6 (2.2)	2 (2.2)	0 (0)

^a^ Data not recorded in 15 cases.

^b^ Data not recorded in 223 cases.

^c^ Data not recorded in 208 cases.

^d^ In multiple assailant assaults, the description of the individual designated as the primary assailant is the one included for analysis.

^e^ Data not recorded in 593 cases.

^f^ Data not recorded in 376 cases.

^g^ Data not recorded in 390 cases.

**TABLE 2:** Incident characteristics stratified by assailant‐victim/survivor relationship.

	Acquaintance <24 h (*n* [%])	Acquaintance >24 h (*n* [%])	Family member (*n* [%])	Intimate partner (*n* [%])	Ex‐intimate partner (*n* [%])	Friend (*n* [%])	Stranger (*n* [%])	Person in authority (*n* [%])	Other (*n* [%])	Unknown (*n* [%])	*P*‐value (*n* [%])
Location of incident^a^											
Home	127 (14.0)	252 (21.7)	113 (49.6)	199 (59.1)	174 (46.9)	204 (26.3)	147 (10.7)	22 (23.4)	15 (20.8)	39 (11.7)	<0.001
Assailant's home	244 (27.0)	344 (29.7)	66 (28.9)	74 (22.0)	99 (26.7)	232 (29.9)	228 (16.7)	15 (16.0)	18 (25.0)	9 (2.7)	
Other indoors	304 (33.6)	261 (22.5)	29 (12.7)	36 (10.7)	42 (11.3)	164 (21.2)	375 (27.4)	43 (45.7)	23 (31.9)	76 (22.9)	
Field/park	48 (5.3)	76 (6.6)	3 (1.3)	9 (2.7)	11 (3.0)	59 (7.6)	103 (7.5)	1 (1.1)	1 (1.4)	22 (6.6)	
Other outdoors	125 (13.8)	139 (12.0)	7 (3.1)	10 (3.0)	19 (5.1)	73 (9.4)	369 (27.0)	2 (2.1)	8 (11.1)	79 (23.8)	
Taxi	6 (0.7)	0 (0)	1 (0.4)	0 (0)	0 (0)	2 (0.3)	47 (3.4)	1 (1.1)	3 (4.2)	2 (0.6)	
Vehicle	34 (3.8)	70 (6.0)	4 (1.8)	4 (1.2)	18 (4.9)	28 (3.6)	72 (5.3)	5 (5.3)	2 (2.8)	7 (2.1)	
Other	9 (1.0)	9 (0.8)	4 (1.8)	3 (0.9)	5 (1.3)	6 (0.8)	12 (0.9)	5 (5.3)	2 (2.0.8)	4 (1.2)	
Unsure	7 (0.8)	9 (0.8)	1 (0.4)	2 (0.6)	3 (0.8)	7 (0.9)	15 (1.1)	0 (0)	0 (0)	94 (28.3)	
Concern for a drug‐facilitated sexual assault^b^											
Yes	171 (20.2)	166 (15.4)	26 (12.9)	31 (9.8)	35 (9.8)	101 (13.8)	255 (20.1)	12 (15.0)	6 (9.4)	128 (40.3)	<0.001
Unsure	145 (17.2)	169 (15.7)	24 (11.9)	21 (6.7)	41 (11.5)	117 (16.0)	85 (17.9)	6 (7.5)	52 (81.2)	85 (26.7)	
No	529 (62.6)	742 (68.9)	280 (75.1)	263 (83.5)	280 (78.7)	514 (70.2)	784 (62.0)	62 (77.5)	6 (9.4)	105 (33.0)	
Drug use in the 24 h prior to incident^c^											
Yes	199 (22.6)	224 (19.9)	26 (12.4)	40 (12.6)	62 (17.6)	129 (17.1)	211 (15.9)	6 (7.3)	8 (11.8)	46 (13.3)	<0.001
No	677 (76.8)	898 (79.8)	184 (87.6)	278 (87.4)	295 (82.4)	620 (82.6)	1105 (83.3)	76 (92.7)	60 (88.2)	293 (85.2)	
Unsure	5 (0.6)	3 (0.3)	0 (0)	0 (0)	1 (0.3)	2 (0.3)	10 (0.8)	0 (0)	0 (0)	5 (1.5)	
Alcohol use in the 24 h prior to incident^d^											
Nil	148 (16.9)	343 (30.5)	123 (58.9)	183 (57.9)	175 (49.2)	223 (29.9)	303 (22.8)	56 (68.3)	32 (47.8)	51 (15.0)	<0.001
≤6 standard drinks^e^	206 (23.5)	8 (9.8)	31 (14.8)	61 (19.3)	76 (21.3)	160 (21.4)	296 (22.2)	8 (9.8)	16 (23.9)	92 (27.1)	
>6 standard drinks^e^	505 (57.8)	17 (20.7)	52 (24.9)	67 (21.2)	103 (28.9)	346 (46.4)	715 (53.7)	17 (20.7)	19 (28.4)	193 (56.8)	
Unsure	16 (1.8)	1 (1.2)	3 (1.4)	5 (1.6)	2 (0.6)	17 (2.3)	17 (1.3)	1 (1.2)	0 (0)	4 (1.2)	

^a^ Data not recorded in 301 cases.

^b^ Data not recorded in 689 cases.

^c^ Data not recorded in 479 cases.

^d^ Data not recorded in 495 cases.

^e^ Standard drink: 10 g of pure alcohol or one measure of spirits (35.5 mL).

Where the assailant was a stranger or someone known for a duration of less than 24 h, the location was significantly more likely to be outdoors (OR 2.28, 95% CI 2.01–2.60 *P* < 0.001). If the assailant was known to the victim, the incident was significantly more likely to take place in the victim's home. (OR 2.90, 95% CI 2.51–3.35, *P* < 0.0001).

As regards the timing of the incident, 5082 (85.6%) were between 17:00 and 07:59, with 3540 (59.5%) occurring between midnight and 07:59.

### 3.4 Body injuries (genital and extra‐genital)

Significant associations were found between the presence of bodily injuries and some incident details, including alcohol consumption or drug use disclosure, time of attendance to SATU and the location of the incident. These are shown in Table 3.

**TABLE 3:** Significant associations of incident details with the presence of bodily injury.

	Bodily injury		
	Relative risk	Confidence interval	*P*‐value
Adult (≥18 years)	1.26	1.08–1.47	0.003
Women who reported the crime to police	2.27	2.00–2.57	<0.001
Women who attended within the first 24 h	2.07	1.85–2.32	<0.001
Women who disclosed alcohol consumption in 24 h prior to incident	1.48	1.30–1.68	<0.001
Women who disclosed drug use in 24 h prior to incident	1.17	1.00–1.36	0.040
Reported concern for drug‐facilitated sexual assault	1.37	1.21–1.54	<0.001
The location of the incident was outdoors	1.37	1.20–1.57	<0.001
Perpetrator being a stranger/<24 acquaintance	1.13	1.01–13	0.032
The use of a weapon	1.66	1.35–2.04	<0.001

### 3.5 Multiple assailant incidents

Multiple assailant assaults accounted for 435 (7.3%) of attendances. Victims of incidents involving more than one assailant were significantly more likely to have used drugs in the 24 h prior to the incident (OR 1.85, 95% CI 1.47–2.33, *P* < 0.0001), for the perpetrators to be strangers and/or people they met within the last 24 h (OR 2.13, 95% CI 1.75–2.60, *P* < 0.0001) and for the location of the incident to occur outdoors (OR 1.53, 95% CI 1.22–1.91, *P* < 0.001).

## 4 DISCUSSION

### 4.1 Main findings

This study, one of the largest on female attendances to a national SATU network, offers detailed insights into demographics, incident details, and circumstances. Most attendees were young women, predominantly 18 years and older, many of whom were full‐time students. Forensic examinations were the primary reason for attendance, underscoring the network's key role in evidence collection. The study also identified factors linked to a higher risk of injury detection, such as prompt post‐incident attendance, being over 18 years, outdoor incidents, the incident being perpetrated by a stranger, and alcohol or drug use in the preceding 24 h.

### 4.2 Attendance details

During the study period, the number of female attendances increased by 21%. A transient decrease in numbers was observed in 2020, coinciding with the COVID‐19 pandemic; however, despite significant curtailment of social activities, sexual assault disclosures still occurred during these restrictions.^10^ With increased attendances, there are instances where a person cannot be seen within 3 h, as another case may be ongoing and each unit only has a single forensic suite. This results in delay in obtaining forensic swabs, which also means that people are advised to defer activities such as eating, washing, and toileting for a longer period of time. It also means that access to other aspects of SATU care (e.g. psychological support, provision of medications) may be similarly delayed. In relation to the increase in attendances year on year, it may be that the incidence of sexual assault itself is not rising, but rather that people are more likely to disclose and seek support. In some respects, this is encouraging, as it indicates that more women are accessing appropriate post‐assault care, including emergency contraception and psychological support. There are a range of care options offered by the SATU services, and this study highlights that all pathways are regularly accessed, underpinning the importance of offering patient choice. Over 10% of women opted for a forensic examination without immediate police involvement, opting instead for evidence storage. Offering an evidence storage examination option after sexual assault is crucial for several reasons. Research has shown that delayed reporting allows victims time to decide whether to involve the police, leading to increased reporting rates.^11^, ^12^ Furthermore, delayed reporting options, such as storing forensic evidence without immediate police notification, can preserve crucial evidence that may be lost if reporting is rushed, potentially aiding in the comprehensive detection of the crime, even if reporting to the police is delayed.^13^ This approach aligns with the need for a trauma‐informed response to sexual assault cases, acknowledging the emotional distress and complexities victims may face during the reporting process.

A previous empirical analysis focusing on European regions revealed a positive association between rape and unemployment, with female unemployment being a significant factor in explaining this relationship.^14^ With almost a quarter of female attenders being unemployed at the time of attendance, our data support this finding.

Our data also show that our SATU population is racially diverse, and this should encourage us to further develop culturally sensitive care approaches to allow all patients to feel comfortable and supported to discuss sexual trauma.^15^ This finding also highlights potential language barrier issues and reiterates the sustained need for accessible interpreter services, which are available at the SATU network.

As regards the age profile of female patients, the highest proportion was in the 18–24 years cohort, which would be in keeping with previous studies.^16^ However, all age cohorts were represented, which reiterates the fact that sexual assault has no age barrier, and thus education, mitigation, and response strategies should not solely be designed for or confined to a specific age group.

### 4.3 Alcohol/drug‐use

Alcohol consumption in the 24 h preceding the incident was disclosed by all age groups, with 79.3% of those between 18 and 24 years disclosing it. This was in contrast to those aged 14–16 years and 55–69 years, where the disclosure rates were 50% and 50.6%, respectively. Alcohol ingestion is frequently blamed as being associated with risk of sexual assault^17^; however, in certain age groups, more than half the female population had not consumed any alcohol. This challenges the stereotype that alcohol is a significant factor in most sexual assaults.^18^

Similarly, our data have shown that the disclosure of drug use in the 24 h preceding the incident is most prevalent in the 25–34 years age bracket, at 22.5%. Several factors may contribute to this situation. The victim might be a habitual drug user, potentially making them inherently vulnerable; prior research indicates that individuals who have multiple vulnerabilities, including drug use, face a higher risk of sexual assault.^19^ Additionally, the person may have consumed drugs before the assault, becoming incapacitated due to intoxication, which the assailant then exploits.

### 4.4 Assailant–victim relationship

It is apparent from the data that assailant–victim relationship significantly affects the location of the incident. In those where the assailant(s) was a stranger or not known, the assault was more likely to occur outdoors, which is consistent with previous studies.^20^, ^21^ Similarly, where the assailant was known to the victim, the assault was significantly more likely to occur in the victim's home. This is likely as a result of a pre‐existing relationship or trust the victim may have in the assailant.

### 4.5 Presence of injury

Regarding the presence of bodily injury, our findings indicate that patients who did not know their assailant are significantly more likely to have sustained an injury during the incident, which is in keeping with previous research.^22^ This finding could be as a result of the relationship of the assailant to the victim, where violence may be more likely to occur when the assailant is not known to the victim. Our data have also shown that assaults by a stranger or recent acquaintance (less than 24 h) are more often perpetrated against adults than against younger women.

Our study also found that the disclosure of alcohol or drug consumption within the 24 h preceding the incident significantly increases the incidence of bodily injury. This suggests two possibilities: the injury may have been caused solely by the assault, or it may have been exacerbated by intoxication‐related factors (e.g., as a result of a fall or because the sedative effect of alcohol makes a person more vulnerable to injury). It is also important to acknowledge that injuries of unknown origin are included in our data, and therefore some injuries may not be directly attributable to the assault itself.

### 4.6 Multiple assailant assault

Multiple assailant assaults accounted for 7% of attendances and were significantly more likely to occur outdoors. This aligns with a previous review indicating that many victims were approached and assaulted outdoors.^23^ Our data also showed that victims of multiple assailant offenses were more likely to have consumed alcohol or drugs in the 24 h before the incident. Victims might have voluntarily consumed these substances or been encouraged by offenders to promote sexual compliance. This highlights the need for social education on the impact of substance misuse and its association with enhanced vulnerability.

### 4.7 Strengths and limitations

Our study has notable strengths, including the use of data from the entire national SATU network in the Republic of Ireland, providing a comprehensive overview of sexual assault cases and enhancing generalizability and transferability. With a large sample size of 5942 female attendances over a 7‐year period, the study increases statistical power and reliability. Additionally, the inclusion of extensive demographic, attendance, and incident details offers an in‐depth understanding of sexual assault cases. However, the study has limitations: it is cross‐sectional and cannot establish causality, relies on paper medical records which may introduce errors, and uses self‐reported data on drug and alcohol use, potentially leading to recall bias and under‐reporting. Furthermore, it only includes SATU attendances, leading to selection bias and possible underestimation of the incidence and characteristics of sexual assault.

## 5 CONCLUSION

This study is one of the largest studies detailing female attendances at a national SATU network. It provides comprehensive insights into the demographic characteristics, incident details, and circumstances of female attendances at the national SATU network over a prolonged period and endorses the importance of having accessible and available care, free of charge and around the clock. The majority of attendances were by young women, predominantly aged 18 years and above, with a significant proportion engaged in full‐time education. Forensic examinations were the primary reason for attendance, reflecting the network's critical role in evidence collection and legal processes. This study also identified specific factors associated with a higher risk of injury detection, such as immediate attendance post‐incident, being over 18 years old, the incident occurring outdoors, the incident being perpetrated by a stranger, as well as the disclosure of alcohol and/or drug consumption prior to the incident. These findings may help to inform both preventive measures and clinical care approaches.

## AUTHOR CONTRIBUTIONS

D.K., K.F., M.E., and N.M. conceptualized the idea for the study. D.K., J.W., C.P., and D.R. were involved in data curation and synthesis of this data. D.K. wrote the first draft and D.K., K.F., M.E., A.H., N.M., and C.P. were involved in reviewing, offering contributions, and finalizing the manuscript. K.F. and M.E. supervised the study.

## FUNDING INFORMATION

None.

## CONFLICT OF INTEREST STATEMENT

The authors have no conflicts of interest.

## Data Availability

Research data are not shared.
